# Beyond the abscess: *Klebsiella pneumoniae* liver abscess combined with bloodstream infection

**DOI:** 10.3389/fcimb.2025.1698703

**Published:** 2026-01-12

**Authors:** Zhihui Guan, Feifei Shao, Haopeng Wu, Lingmin Zhou, Juan Chen, Feizhen Song, Lanxin Cao, Jinming Luo, Wei Cui, Xiaorong Xiao, Gensheng Zhang, Cheng Zheng

**Affiliations:** 1 Department of Critical Care Medicine, the First People’s Hospital of Taizhou, Taizhou, Zhejiang, China; 2 Department of Emergency Medicine, the First People’s Hospital of Taizhou, Taizhou, Zhejiang, China; 3 Department of Critical Care Medicine, the Affiliated Xiangshan Hospital of Wenzhou Medial University, Ningbo, Zhejiang, China; 4 Department of Critical Care Medicine, Shengzhou People’s Hospital, Shaoxing, Zhejiang, China; 5 Department of Critical Care Medicine, Second Affiliated Hospital, Zhejiang University School of Medicine, Hangzhou, Zhejiang, China; 6 Key Laboratory of Multiple Organ Failure (Zhejiang University), Ministry of Education, Ningbo, Zhejiang, China; 7 Taizhou Municipal Hospital (Taizhou University Affiliated Municipal Hospital), School of Medicine, Taizhou University, Taizhou, Zhejiang, China; 8 Key Laboratory of Sepsis of Taizhou, Taizhou, Zhejiang, China

**Keywords:** clinical characteristics, *Klebsiella pneumoniae* bloodstream infection, *klebsiella pneumoniae* liver abscess, outcomes, risk factor

## Abstract

**Objective:**

The clinical characteristics of *Klebsiella pneumoniae* liver abscess (KPLA) and *Klebsiella pneumoniae* bloodstream infection (KP-BSI) are often reported, while the risk factors for KPLA combined with KP bloodstream infection (KPLA/KP-BSI) among KPLA are largely unknown. Therefore, this study aimed to investigate the clinical characteristics, risk factors, and outcomes of patients with KPLA complicated by KP-BSI.

**Methods:**

A retrospective study from May 2013 to October 2020 at a tertiary hospital compared KPLA patients with and without KP-BSI, analyzing clinical data.

**Results:**

Among all liver abscess cases during the study period, *Klebsiella pneumoniae* was the most common pathogen, accounting for 76.0% of isolates. Of 233 KPLA patients, 68.7% were male with a median age of 60.5 years. KPLA/KP-BSI occurred in 27.9%. Patients with KPLA/KP-BSI had higher male prevalence, abdominal surgery history, and higher APACHE II, SOFA, and CCI scores (p<0.05). Logistic regression showed SOFA score ≥ 2 (aOR 3.326) was a risk factor for KPLA/KP-BSI, while liver abscess size > 10 cm reduced the risk (aOR 0.144). KPLA/KP-BSI was associated with worse outcomes, including higher septic shock, acute kidney injury, transfusion rates, organ dysfunction, pneumonia, longer hospital stays, and higher mortality (all p<0.05).

**Conclusion:**

Nearly one-third of patients with KPLA have concurrent KP-BSI. A SOFA score ≥2 is an independent risk factor, whereas abscess diameter >10 cm is protective. KPLA/KP-BSI is associated with significantly higher rates of septic shock, organ dysfunction, and in-hospital mortality, warranting heightened clinical attention.

## 1 Introduction

The liver abscess (LA) is a potentially lethal infectious disease with various etiologies, including bacterial (pyogenic), amoebic, and fungal. Pyogenic liver abscess (PLA) is a potentially life-threatening infection, accounting for approximately 80% of all liver abscesses worldwide (Shi et al., 2018). Compared with Europe and the United States, the incidence of pyogenic LA in Asia is remarkably increased, reaching 17.6 per 100,000 population (Deschler and Lübbert, 2006). Risk factors for amoebic LA include travel to endemic areas and immunosuppression, while fungal LA, such as those caused by *Aspergillus*, are associated with neutropenia, prolonged antibiotic use, or organ transplantation (Herrera et al., 2025). Recent findings from Sweden shown an increasing incidence of bacterial liver abscesses over time, partly due to the aging population and immunosenescence (Båve and Bergquist, 2021). Moreover, the diagnosis of LA presents considerable challenges due to its non-specific symptoms, such as fever, right upper quadrant abdominal pain, or vomiting, complicating the treatment decisions (Serraino et al., 2018). Treatment of pyogenic LA relies on drainage of the abscess and effective antimicrobial therapy (Lam et al., 2018). Despite this, the mortality rate of pyogenic LA is still as high as 19.0 percent (Hsu et al., 2020), with variations based on regional medical practices and patient comorbidity burden. Therefore, pyogenic LA is still a serious disease in clinical practice.

Many common pathogens are identified to be involved in the pathogenesis of pyogenic LA including *Streptococcus*, anaerobes, and gram-negative bacteria such as *Escherichia coli (*Morris and Cerceo, 2020). However, *Klebsiella pneumonia* (KP) has gradually become the most common pathogen of pyogenic LA recently, especially in Asia (Rahim et al., 2019). Intestinal colonization and subsequent translocation *via* the portal venous system are proposed mechanisms leading to hepatic seeding and abscess formation (Chen et al., 2023). An analysis showed that gram-negative bacteria account for 70% of etiology in Chinese pyogenic LA patients, with *Klebsiella* being the most common pathogen (54%) (Zhang et al., 2017). The mortality rate for KP liver abscess (KPLA) shows significant regional variance, ranging from 3% to 42% (Zhang et al., 2017). However, current research provides almost no insights into KPLA with concurrent bloodstream infections, a condition with a worse prognosis and higher complication rates. In addition, patients with KPLA who develop sepsis experience a higher incidence of clinical complications, encompassing debilitated state, diarrhea, fatty liver, chronic renal insufficiency, and hepatic dysfunction, than those without sepsis (Zhang et al., 2019). These patients also exhibit elevated rates of metastatic infections, particularly lung metastases (Zhang et al., 2019). It is evident that, despite growing recognition of the clinical impact of KPLA, no such study on KPLA complicated with *Klebsiella pneumoniae* bloodstream infection (KP-BSI) has been reported yet. This gap reveals a significant deficiency in our understanding of this severe complication, making the development of effective treatment strategies challenging.

Hence, our study aims to bridge this considerable knowledge gap, marking the first comprehensive investigation of KPLA patients with KP-BSI (KPLA/KP-BSI), to reveal its epidemiology, clinical characteristics, risk factors, and its outcomes. The findings of this study are anticipated to have some implications for further early recognizing, improving care, and reducing mortality rates of patients with KPLA/KP-BSI.

## 2 Subjects and methods

### 2.1 Subjects

The diagnosis and treatment processes of these patients were retrospectively reviewed at the Second Affiliated Hospital of Zhejiang University. Ethical approval for this study was obtained from the Ethics Committee at the aforementioned institution (Approval No. 2021-943). Given the retrospective nature of the study and the absence of potential risks to the patients, informed consent was waived.

### 2.2 Inclusion and exclusion criteria

The study’s inclusion criteria were as follows: (1) patients aged 18 years or older; (2) hospitalization primarily due to primary pyogenic liver abscess (PLA, defined as idiopathic without underlying malignancy or procedure) with visualized lesions found only in the liver through image examinations including CT, MRI, or ultrasound; (3) diagnosis of KPLA; (4) availability of complete diagnosis and clinical data. Exclusion criteria for the study included: (1) liver abscess caused by other organisms like fungi, *Mycobacterium tuberculosis*, or amoeba; (2) secondary liver abscesses (arising from malignancy, trauma, or biliary obstruction); (3) history of liver and kidney transplantation; (4) discharge within 24 hours; (5) incomplete or missing records.

Patients diagnosed with KPLA had to meet all the following criteria (Siu et al., 2012): (1) clinical symptoms such as chills, high fever, right upper abdominal pain, nausea, or vomiting; (2) imaging examinations showing cystic lesions in the liver through abdominal ultrasound, CT, or MRI; (3) *klebsiella pneumoniae* was confirmed from culture of liver abscess fluid.

Patients diagnosed with KPLA-BSI had to meet the CDC/NHSN standards for bloodstream infections, that was to say, the same drug-sensitive *Klebsiella pneumoniae* in blood cultures along with a KPLA diagnosis (Bloodstream infection event (Central line-associated bloodstream infection and non-central line associated bloodstream infection)).

### 2.3 Bacterial culture and antibiotic sensitivity test

The puncture drainage fluid of the liver abscess and blood samples were collected and injected into the aerobic and anaerobic culture bottles. Through the Bac/Alert 3D full-automatic blood culture instrument, the positive samples were identified and then subcultured for further identification.

For the drug susceptibility test, a suspension was adjusted to 0.5 Maxwell turbidity, identified, and analyzed using the MicroScanwalkAway-96 Bacterial Identification and Drug Susceptibility Analyzer (Siemens, USA). The findings were interpreted using the 2019 version of the Clinical and Laboratory Standards Institute standards (WM, 2019).

### 2.4 Prognostic assessment and data collection

To assess the prognosis of patients, we employed a combination of the Sequential Organ Failure Assessment (SOFA) scale, the Acute Physiology and Chronic Health Evaluation (APACHE II) scores, and the age-adjusted Charlson Comorbidity Index (aCCI) scores (Charlson et al., 1994). The significance of using the aCCI lies in its ability to assess the burden of comorbidities and predict mortality, thereby providing valuable insights into the severity of the patient’s condition. These tools collectively offer a comprehensive evaluation of a patient’s comprehensive health status, severity of comorbidities, and potential for mortality.

Data collection was conducted through the electronic medical record system, including demographic characteristics (such as gender, age, APACHE II score), information on disease history, clinical symptoms, laboratory and imaging examinations, microbial data, treatments, complications, and outcomes.

### 2.5 Statistical analysis

All statistical analyses were carried out using the SPSS 24.0 Statistics software (IBM Corp, Armonk, NY, USA). Continuous variables with normal distribution were presented as means ± SD and the *t-*test was used. Continuous variables with non-normal distribution were presented with median and interquartile range (IQR), while comparisons between groups were carried out using the Mann-Whitney U test. Comparison of categorical variable between two groups was carried out using the Pearson χ^2^ or Fisher’s Exact Test, and a p-value of <0.05 was considered statistically significant. Multivariate logistic regression was used to determine the independent risk factors, and a double-tailed p-value of <0.05 was considered to be statistically significant.

## 3 Results

### 3.1 Epidemiological Analysis of KPLA/KP-BSI

**Figure 1:**
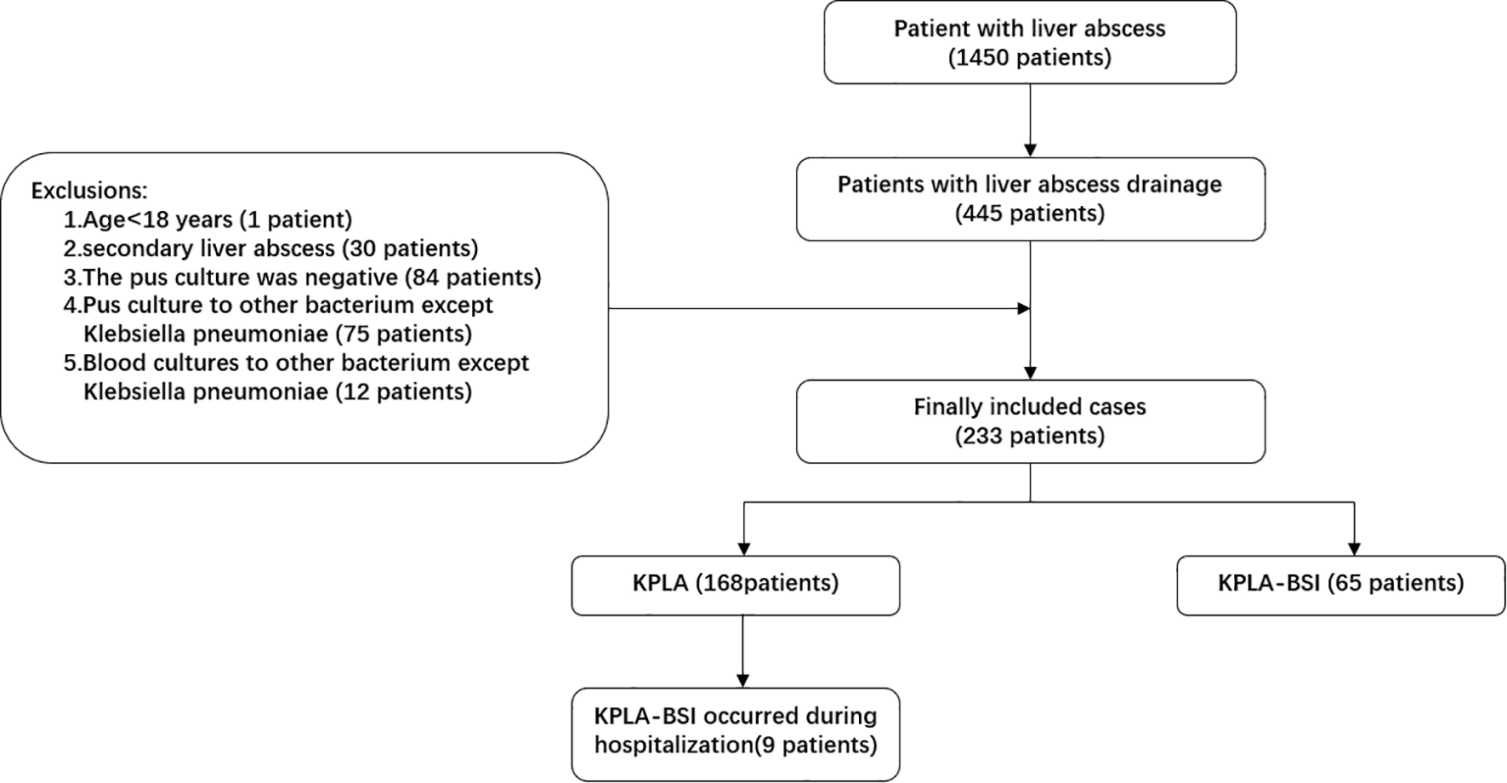
Flowchart of participant enrollment in the present study.

**Figure 2:**
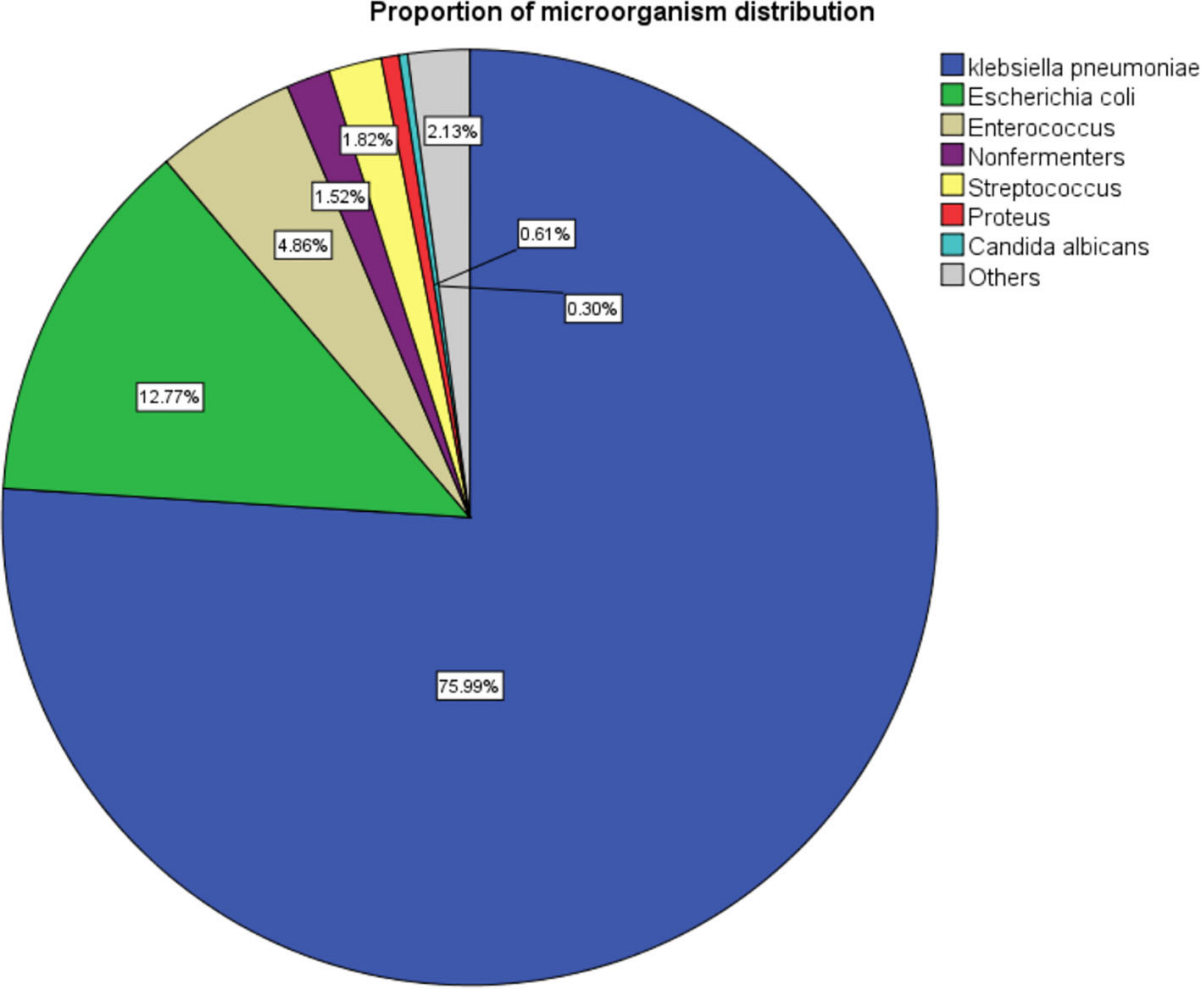
Microbial etiology of liver abscess cases registered from May 2013 to October 2020.

A total of 233 patients with KPLA were finally included in this study, comprising 168 patients (72.1%) with KPLA alone and 65 patients (27.9%) with KPLA/KP-BSI (**Figure 1**). To further investigate the incidence trend of KPLA alone and KPLA/KP-BSI among the enrolled patients between 2013 and 2020, we observed a gradual increase in both KPLA alone and KPLA/KP-BSI throughout these years. Upon retrospectively analyzing patients diagnosed with liver abscesses, we discovered that the most common causative pathogen was *Klebsiella pneumoniae* (KP), accounting for 76.0% of all liver abscess cases reviewed (**Figure 2**).

### 3.2 Demographic and clinical data

**Table 1** shows the differences in demographic and clinical characteristics between the two groups. In comparison with KPLA alone, patients with KPLA/KP-BSI had higher proportions of male cases and abdominal surgery history, and displayed higher scores of SOFA, APACHE II, and aCCI (all P<0.05).

**Table 1:** Demographic and clinical characteristics of the patients with KPLA alone or KPLA/KP-BSI.

Characteristics	Total (233 cases)	KPLA alone (168 cases)	KPLA/KP-BSI (65 cases)	P value
Age (years), mean (± SD)	60.5 ± 14.5	60.0 ± 15.3	61.6 ± 12.2	0.425
Gender				
Male, n (%)	160 (68.7%)	109 (64.9%)	51 (78.5%)	**0.045**
Female, n (%)	73 (31.3%)	59 (35.1%)	14 (21.5%)	**0.045**
Personal history				
Smoking habit	75 (32.2%)	49 (29.2%)	26 (40.0%)	0.112
Drinking habit	82 (35.2%)	55 (32.7%)	27 (41.5%)	0.268
Previous history of liver abscess	6 (2.6%)	3 (1.8%)	3 (4.6%)	0.446(b)
Symptoms				
Hyperpyrexia (T>39°C)	172 (73.8%)	122 (72.6%)	50 (76.9%)	0.503
Abdominal pain	80 (34.3%)	61 (36.3%)	19 (29.2%)	0.307
Nausea	50 (21.5%)	37 (22.0%)	13 (20.0%)	0.736
Vomiting	34 (14.6%)	23 (13.7%)	11 (16.9%)	0.531
Diarrhea	13 (5.6%)	6 (3.6%)	7 (10.8%)	**0.067**(b)
Debilitated state	178 (76.4%)	125 (74.4%)	53 (81.5%)	0.25
Underlying conditions				
Diabetes mellitus	99 (42.5%)	71 (42.3%)	28 (43.1%)	0.91
Hypertension	76 (32.6%)	54 (32.1%)	22 (33.8%)	0.804
Fatty liver	42 (18.0%)	27 (16.1%)	15 (23.1%)	0.212
Cholelithiasis	45 (19.3%)	33 (19.6%)	12 (18.5%)	0.838
Viral hepatitis	5 (2.1%)	3 (1.8%)	2 (3.1%)	0.916(b)
Non-hematological solid tumors	29 (12.4%)	17 (10.1%)	12 (18.5%)	0.084
Chronic renal insufficiency	4 (1.7%)	1 (0.6%)	3 (4.6%)	0.120 (b)
Abdominal surgery history	59 (25.3%)	34 (20.2%)	25 (38.5%)	**0.004**
COPD or asthma	15 (6.4%)	11 (6.5%)	4 (6.2%)	1.000(b)
Chemoradiotherapy	4 (1.7%)	1(0.6%)	3(4.6%)	0.120(b)
Cerebrovascular accident	4 (1.7%)	3(1.8%)	1(1.5%)	1.000(b)
Liver cirrhosis	3 (1.3%)	1(0.6%)	2(3.1%)	0.189(a)
aCCI (IQR)	4.0 (2.0,5.0)	3.0 (2.0,4.0)	4.0 (3.0,5.0)	**0.04**
APACHE II	9.0 (6.0,11.0)	8.0 (6.0,11.0)	11.0 (8.0,13.0)	**<0.001**
SOFA	2.0 (0.0,4.0)	1.0 (0.0,3.0)	5.0 (2.0,6.0)	**<0.001**

(a) Fisher’s exact test, (b) χ^2^ test with continuity correction. Bold values indicate statistical significance (p < 0.05).

COPD, chronic obstructive pulmonary disorder; CCI, Charlson Comorbidity Index; APACHE, acute physiology and chronic health evaluation; SOFA, sequential organ failure assessment; IQR, interquartile range.

### 3.3 Imaging findings and laboratory tests

When comparing imaging indicators (**Table 2**), we found that the proportion of abscesses with a diameter greater than 10 cm in the KPLA group was significantly higher than that in the KPLA/KP-BSI group (18.1% vs. 6.5%, p=0.028).

**Table 2:** Imaging findings and laboratory findings of patients with KPLA alone or KPLA/KP-BSI.

Characteristics	Total (233 cases)	KPLA alone (168 cases)	KPLA-BSI (65 cases)	P value
Liver imaging				
Abscess location (222 cases)				
Left lobe	39 (16.7%)	28 (16.7%)	11 (16.9%)	0.962
Right lobe	170 (73.0%)	125 (74.4%)	45 (69.2%)	0.425
Both lobes	22 (9.4%)	15 (8.9%)	7 (10.8%)	0.667
Quantity				0.126
1	187 (80.3%)	139 (82.7%)	48 (73.8%)	
≥2	46 (19.7%)	29 (17.3%)	17 (26.2%)	
Abscess size (cm)				
<5cm	50 (22.5%)	31 (19.4%)	19 (30.6%)	0.071
5 - 10cm	141 (62.7%)	102 (62.6%)	39 (62.9%)	0.964
>10cm	33 (14.9%)	29 (18.1%)	4 (6.5%)	0.028*
biological indicator				
Body temperature (°C) (IQR)	39.1 (38.8,39.9)	39.1(39.0,39.8)	39.4(39.0,40.0)	**0.049**
WBC (×10^9^/L) (IQR)	12.2 (9.4,15.9)	12.1 (9.6,15.6)	13.1 (9.6,17.9)	0.073
ANC (×10^9^/L) (IQR)	10.5 (7.6,14.5)	10.3 (7.7,13.9)	11.9 (8.2,16.8)	<0.001
NE (%) (IQR)	86.0 (80.8,91.2)	85.0 (80.1,89.3)	90.5 (85.2,93.6)	**<0.001**
HB (g/L) (IQR)	118.3 ± 18.0	118.0 ± 17.8	119.2 ± 18.6	0.65
HCT (%) (IQR)	35.1 (32.2,38.4)	35.0 (32.2,38.3)	35.0 (31.6,38.5)	0.665
PLT (×10^9^/L) (IQR)	170.0 (98.3,274.0)	174.0 (115.0,295.3)	95.0 (40.3,187.0)	**<0.001**
CRP (mg/L) (IQR)	179.8 (123.2,244.4)	190.4 (123.5,245.6)	182.3 (126.9,250.9)	0.502
PCT (ng/ml) (IQR)	5.5 (0.9,25.0)	2.8 (0.7,17.6)	18.1 (3.9,45.3)	**<0.001**
ALB (g/L) (IQR)	30.9 (26.2,34.5)	32.1 (26.4,35.0)	28.1 (25.2,33.0)	**0.003**
TBil (μmol/L) (IQR)	15.8 (11.5,29.4)	15.9 (11.5,28.1)	18.6 (11.4,36.9)	0.525
ALT (U/L) (IQR)	63.0 (38.5,101.0)	56.5 (32.0,89.8)	69.0 (43.5,110.0)	**0.037**
AST (U/L) (IQR)	52.0 (30.5,98.5)	40.0 (27.0,71.8)	63.0 (37.0,133.5)	**0.001**
ALP (U/L) (IQR)	152.0 (105.0,222.0)	165.5 (115.8,224.8)	129.0 (89.0,210.0)	**0.017**
GGT (U/L) (IQR)	96.0 (58.0,164.0)	112.0 (58.3,169.3)	81.0 (52.5,156.0)	0.21
LDH (U/L) (IQR)	237.0 (191.0,308.0)	233.5 (191.0,273.3)	243.0 (195.0,336.0)	0.216
SCr (μmol/L) (IQR)	67.0 (53.0,92.0)	66.0 (52.8,86.3)	77.0 (55.5,107.0)	0.057
Lactate (mmol/L) (IQR)	1.0 (0.8,1.7)	0.9 (0.7,1.3)	1.6 (1.0,2.6)	**<0.001**
PT (s) (IQR)	14.5 (13.7,15.7)	14.3 (13.6,15.2)	14.9 (13.6,16.2)	**0.017**
APTT (s) (IQR)	39.7 (35.7,43.7)	39.8 (35.7,44.6)	39.5 (35.5,42.6)	0.936
Fib (g/L) (IQR)	6.8 (5.3,7.8)	6.9 (5.5,7.8)	6.5 (4.7,7.6)	0.08
Random blood sugar (mmol/L) (IQR)	9.3 (6.6,13.7)	9.0 (6.6,13.7)	10.0 (7.2,14.3)	0.267

WBC, white blood count; NE, neutrophils; HB, Hemoglobin; HCT, hematokrit; PLT, platelet; CRP, C reactive protein; PCT, procalcitonin; ALB, albumin; TBil, total bilirubin; ALT, Alanine transaminase; AST, Aspartate transaminase; ALP, alkaline phosphatase; GGT, gamma-glutamyltransferase; LDH, lactic dehydrogenase; SCr, serum creatinine; PT, prothrombin time; APTT, activated partial thromboplastin time; Fib, fibrinogen; IQR, interquartile range. Bold values indicate statistical significance (p < 0.05).

In comparison with patients with KPLA alone, laboratory tests revealed that patients with KPLA/KP-BSI exhibited poorer liver functions, as indicated by lower levels of albumin (ALB) (median g/L, 28.1 vs. 32.1, p=0.003) and alkaline phosphatase (ALP) (median U/L, 129.0 vs. 165.5, p=0.017), higher levels of alanine aminotransferase (ALT) (median U/L, 69.0 vs.56.5, p=0.017) and aspartate aminotransferase (AST) (median U/L, 63.0 vs. 40.0, p=0.001), elevated lactic acid levels (median mmol/L, 1.6 vs. 0.9, p<0.001), and prolonged prothrombin time (PT) (median s, 14.9 vs. 14.3, p=0.017). Additionally, higher levels of inflammatory indicators, such as the percentage of neutrophils (median %, 90.5 vs. 85.0, p<0.001) and procalcitonin (median ng/ml, 18.1 vs. 2.8, p<0.001), were observed in patients with KPLA/KP-BSI (**Table 2**). Absolute neutrophil counts (ANC) were calculated from WBC × NE% and were higher in the KPLA/KP-BSI group (median 11.9 ×10^9^/L vs. 10.3 ×10^9^/L, p<0.001).

### 3.4 Antibiotic resistance in groups of KPLA and KPLA/KP-BSI

Antibiotic sensitivity tests showed that amikacin (1.3%) had the lowest resistance (2.1%), sequentially followed by imipenem (2.1%) and meropenem (2.1%). The resistance rate of *Klebsiella pneumoniae* in the abscess drainage fluid to imipenem (6.2% vs. 0.6%, p=0.034), meropenem (6.2% vs. 0.6%, p=0.034), piperacillin/tazobactam (9.2% vs. 1.2%, p=0.009), ciprofloxacin (16.9% vs. 2.4%, p<0.001), levofloxacin (27.7% vs. 5.4%, p<0.001), and aztreonam (9.2% vs. 1.8%, p=0.023), was significantly higher in KPLA/KP-BSI group compared to KPLA group (**Table 3**).

**Table 3:** Antibiotics resistance among *Klebsiella pneumoniae* isolates.

Antibiotics	Total (n=233)	KPLA alone (n=168)	KPLA/KP-BSI (n=65)	P value
Imipenem (168 vs 65) ^c^	5 (2.1%)	1 (0.6%)	4 (6.2%)	0.034 (b)**
Meropenem (168 vs 65) ^c^	5 (2.1%)	1 (0.6%)	4 (6.2%)	0.034 (b)**
Cefoperazone/sulbactam (168 vs 65) ^c^	10 (4.3%)	6 (3.6%)	4 (6.2%)	0.609 (b)**
Piperacillin/tazobactam (168 vs 65) ^c^	8 (3.4%)	2 (1.2%)	6 (9.2%)	0.009 (b)*
Amoxicillin-clavulanic acid (160 vs 65) ^c^	12 (5.3%)	6 (3.8%)	6 (9.2%)	0.183 (b)**
Ampicillin (94 vs 64) ^c^	125 (79.1%)	92 (97.9%)	33 (51.6%)	<0.001***
Ceftriaxone (160 vs 65) ^c^	12 (5.3%)	6 (3.8%)	6 (9.2%)	0.183 (b)**
Cefepime (168 vs 65) ^c^	7 (3.0%)	3 (1.8%)	4 (6.2%)	0.186 (b)**
Cefoxitin (160 vs 65) ^c^	15 (6.7%)	8 (5.0%)	7 (10.8%)	0.201 (b)*
Ceftazidime (168 vs 65) ^c^	11 (4.7%)	6 (3.6%)	5 (7.7%)	0.324 (b)**
Cefazolin (160 vs 65) ^c^	29 (12.9%)	21 (13.1%)	8 (12.3%)	0.868
Ciprofloxacin (168 vs 65) ^c^	15 (6.4%)	4 (2.4%)	11 (16.9%)	<0.001 (b)*
Levofloxacin (168 vs 65) ^c^	27 (11.6%)	9 (5.4%)	18 (27.7%)	<0.001***
Amikacin (167 vs 65) ^c^	3 (1.3%)	1 (0.6%)	2 (3.1%)	0.393 (b)
Aztreonam (168 vs 65) ^c^	9 (3.9%)	3 (1.8%)	6 (9.2%)	0.023 (b)**
SMZ-TMP (168 vs 65) ^c^	22 (9.4%)	12 (7.1%)	10 (15.4%)	0.054
Tigecycline (168 vs 65) ^c^	9 (3.9%)	6 (3.6%)	3 (4.6%)	1.000 (b)
Tobramycin (168 vs 65) ^c^	8 (3.4%)	5 (3.0%)	3 (4.6%)	0.830 (b)
Polymyxin (11 vs 2) ^c^	2 (15.4%)	1 (9.1%)	1 (50.0%)	0.295 (a)
Nitrofurantoin (93 vs 64) ^c^	91 (58.0%)	68 (73.1%)	23 (35.9%)	<0.001***
Gentamicin (160 vs 65) ^c^	6 (2.7%)	4 (2.5%)	2 (3.1%)	1.000 (b)

(a) use fishier test, (b) use chi square test continuity correction; c:the numbers in parentheses represent the total numbers of *Klebsiella pneumoniae* performed susceptibility test. Bold values indicate statistical significance (p < 0.05). *p < 0.05; **p < 0.01; ***p < 0.001.

### 3.5 Multivariable logistic regression of factors for KPLA/KP-BSI

Multivariate logistic regression model analysis showed that the independent risk factor of KPLA/KP-BSI is SOFA score ≥2 (adjusted odds ratio (OR), 3.326; 95% confidence interval (CI), 1.352-0.554), while abscess size greater than 10 cm is a negatively independent risk factor of KPLA/KP-BSI (aOR, 0.144; 95% CI, 0.037-0.554) (**Table 4**).

**Table 4:** Multivariable logistic regression of factors for KPLA/KP-BSI.

Variable	Unadjusted OR (95% CI)	p-Value	Adjusted OR (95% CI)	P-value
Gender	1.972 (1.008,3.857)	0.047	2.178 (0.086,5.357)	0.090
Abdominal surgery history	2.463 (1.318,4.605)	0.005	1.217 (0.502,2.953)	0.664
aCCI	2.050 (1.129,3.721)	0.018	1.641 (0.817,4.761)	0.245
APACHE II	4.078 (2.212,7.518)	<0.001	1.972 (0.817,4.761)	0.131
SOFA	5.610 (2.795,11.261)	<0.001	3.326 (1.352,8.186)	**0.009**
Abscess size (>10cm)	0.312 (0.105,0.927)	0.036	0.144 (0.037,0.554)	**0.005**
Septic Shock	8.816 (3.272,23.755)	<0.001	2.417 (0.591,9.886)	0.219
AKI	3.485 (1.712,7.096)	0.001	1.576 (0.513,4.839)	0.427
Dysfunction of liver	2.583 (1.282,5.205)	0.008	1.613 (0.624,4.169)	0.324
Pneumonia	3.119 (1.671,5.822)	<0.001	2.208 (0.949,5.138)	0.066
Pyogenic pulmonary embolism	5.593 (1.355,23.081)	0.017	2.481 (0.418,14.717)	0.317
Transfusion	6.750 (2.442,18.654)	<0.001	1.603 (0.400,6.419)	0.505

The cut-off criteria for aCCI and sofa were derived by ROC curves. Bold values indicate statistical significance (p < 0.05).

### 3.6 Comparison of complications and prognosis between groups of KPLA alone and KPLA/KP-BSI

The comparison of prognosis and complications between the two groups is shown in **Table 3**. The most common complication among all patients of KPLA was dysfunction of liver (68.2%), followed by pleural fusion (44.6%) and pneumonia (36.9%). In comparison with patients with KPLA alone, patients with KPLA/KP-BSI had more severe complications and poorer outcomes, evidenced by a longer hospitalization length of stay (mean days, 17.7 vs. 13.8, p = 0.029), a higher in-hospital mortality (15.4% vs. 1.8%, p <0.001), and higher occurrence ratios of septic shock (24.6% vs. 3.6%, p <0.001), acute kidney injury (AKI) (30.8% vs. 11.3%, p <0.001), pneumonia (56.9% vs. 29.2%, p <0.001), liver dysfunction (81.5% vs.63.1%, p=0.007) and more need of blood transfusion (20.0% vs.3.6%, p<0.001). Additionally, patients with KPLA/KP-BSI exhibited a higher incidence of pyogenic pulmonary embolism (9.2% vs. 1.8%, p = 0.023) (**Table 5**).

**Table 5:** Comparison of complications and prognosis between groups of KPLA alone and KPLA/KP-BSI.

Characteristics	Total (233 cases)	KPLA alone (168 cases)	KPLA/KP-BSI (65 cases)	P value
Complication				
Septic Shock	24 (10.3%)	6 (3.6%)	16 (24.6%)	**<0.001**
AKI	39 (16.7%)	19 (11.3%)	20 (30.8%)	**<0.001**
ARF	11 (4.7%)	6 (3.6%)	5 (7.7%)	0.183
acute hepatic dysfunction	159 (68.2%)	106 (63.1%)	53 (81.5%)	**0.007**
pleural effusion	104 (44.6%)	69 (41.1%)	35 (53.8%)	0.079
ascites	46 (19.7%)	30 (17.9%)	16 (24.6%)	0.245
pneumonia	86 (36.9%)	49 (29.2%)	37 (56.9%)	**<0.001**
Suppurative portal vein thrombosis	12 (5.2%)	5 (3.0%)	7 (10.8%)	0.100 (a)
Metastatic infections				
Pyogenic pulmonary embolism	9 (3.9%)	3 (1.8%)	7 (9.2%)	**0.023 (b)**
endophthalmitis	5 (2.1%)	4 (2.4%)	1 (1.5%)	>0.999(b)
cerebral abscess	5 (2.1%)	3 (1.8%)	2 (3.1%)	0.916
subphrenic abscess	1 (0.4%)	1 (0.6%)	0 (0.0%)	>0.999(a)
**transfusion**	19 (8.2%)	6 (3.6%)	13 (20.0%)	**<0.001**
Total Hospitalization days (M) (IQR)	14.9 ± 10.8	13.8 ± 9.7	17.7 ± 13.0	**0.029**
Total ICU residence days (M) (IQR)	2.8 (4.0,19.5)	3.5 (1.8,21)	4.5 (3.0,16.5)	0.367(b)
Targeted antibiotic use				
Carbapenems	173 (74.2%)	122 (72.6%)	51 (78.5%)	0.360
Beta-lactamase inhibitors	45 (19.3%)	33 (19.6%)	12 (18.5%)	0.838
third generation cephalosporin	6 (1.7%)	5 (3.0%)	1 (1.5%)	0.873(b)
Cephamycin	5 (2.1%)	4 (2.4%)	1 (1.5%)	>0.999(b)
Latamoxef	3 (1.3%)	3 (1.8%)	0 (0.0%)	0.562(a)
Ornidazole	2 (0.9%)	2 (1.2%)	0 (0.0%)	>0.999(a)
Quinolone	2 (0.9%)	2 (1.2%)	0 (0.0%)	>0.999(a)
Prognosis				
Condition improved	220 (94.4%)	165 (98.2%)	55 (84.6%)	**<0.001**
Death	13 (5.6%)	3 (1.8%)	10 (15.4%)	**<0.001(b)**

(a) use fishier test, (b) use chi square test continuity correction. Bold values indicate statistical significance (p < 0.05).

AKI, acute kidney injury,AFR, acute respiratory failure.

## 4 Discussion

In the present study, we found that 27.9% of KPLA patients were complicated with KP-BSI. The SOFA score ≥ 2 was an independent risk factor for KPLA/KP-BSI, and larger liver abscesses (>10 cm) are less likely to be complicated with bloodstream infections. In addition, patients with KPLA/KP-BSI were associated with poorer outcomes in comparison with KPLA alone. These results indicate that KPLA/KP-BSI is still a severe clinical issue, highlighting the need for early detection and targeted management. Our 27.9% KP-BSI rate aligns with Zhang et al. (2019) [10], who reported 20-30% in southeastern China, but higher than Western studies (~15%) (Serraino et al., 2018).

This incidence may be influenced by host factors and infection severity, as evidenced by our findings on SOFA scores. We found that patients with a SOFA score greater than 2 are more likely to have concurrent bloodstream infections, which may be related to the following factors: 1) Inflammation and systemic response. KPLA/KP-BSI can lead to a systemic inflammatory response syndrome (SIRS), resulting in multi-organ dysfunction. A SOFA score of ≥2 could reflect a severe systemic inflammatory response due to the infection, leading to multiple organ damage. The release of inflammatory mediators such as cytokines and chemokines may exacerbate tissue damage and organ dysfunction, further increasing the SOFA score (Walden et al., 2010). 2) Microcirculatory disturbance and infection spread. KP-BSI indicates that pathogens have entered the bloodstream from a localized infection site, like a liver abscess (Hoh et al., 2019), potentially causing systemic infection and microcirculatory disturbances. These disturbances can lead to inadequate perfusion of critical organs, causing a decline in organ functions. The bloodstream infection exacerbates the toxic effects of local infection, potentially leading to further deterioration of organ function. 3) Immune response and infection persistence. The status of a patient’s immune system may affect the SOFA score. An immunosuppressed or immunodeficient state could make it easier for multi-organ dysfunction to occur (Kotsaki and Giamarellos-Bourboulis, 2012). Persistent infection pressure could provoke an exaggerated body response, such as an overactive immune response, further increasing the SOFA score (Thakar et al., 2015). 4) Infection control and organ support. Patients with higher SOFA scores may require more supportive treatments, such as respiratory support, vasopressor drugs, or renal replacement therapy, all of which also reflect the severity of the condition (Zampieri et al., 2019). Poor therapeutic responsiveness and inadequate infection control can keep the SOFA score high, reflecting the challenges in treating KP-BSI.

We found that patients with liver abscesses with a diameter greater than 10 cm are less likely to concurrent bloodstream infections (KP-BSI), which may be related to the following factors: 1) Physical characteristics of the abscess. Larger abscesses may have more distinct physical boundaries, which could limit the spread of bacteria into the bloodstream. The presence of more necrotic tissue and pus in larger abscesses might create a physical barrier that inhibits bacterial growth and dissemination. 2) Variations in immune response. Larger abscesses could elicit a stronger local immune response, with increased recruitment of inflammatory cells and immune mediators, which might help contain the infection locally. An intense immune response could form a more effective defensive barrier around larger abscesses, preventing bacteria from entering the bloodstream (Syed and Tellez Watson, 2022). 3) Timeliness of treatment intervention. Large abscesses might be more readily detected and diagnosed clinically, leading to more timely treatment interventions. Prompt treatment can effectively control the infection and reduce the chances of bacteria entering the bloodstream (Tan et al., 2005). 4) Treatment for larger abscesses might require more aggressive approaches, such as drainage or surgery, which could directly reduce the risk of bloodstream infections (Hussain et al., 2007). 5) Formation of bacterial biofilms. In larger abscesses, bacteria might form biofilms, which can protect them from the host’s immune system and antibiotics, but might also limit their ability to spread (Chen et al., 2020). 6) Differential monitoring and management. Patients with large abscesses might be subject to closer monitoring and management, which could include more frequent laboratory tests and imaging studies, allowing for earlier detection and management of potential bloodstream infections. While there is limited literature examining the relationship between abscess size and KPLA-BSI, these hypotheses are grounded in our clinical experience and warrant further investigation.

The poorer prognosis of KPLA/KP-BSI compared to KPLA alone can be substantiated through several key aspects: 1) Increased severity of infection. The presence of KP-BSI indicates a more severe and widespread infection than isolated KP-LA. This severity is often reflected by higher SOFA scores (**Table 1**), indicating more profound organ dysfunction, which is associated with worse outcomes. Biomarkers of infection and inflammation, such as procalcitonin (PCT) (Kikuchi et al., 2023) (**Table 2**), are typically elevated in KPLA/KP-BSI, suggesting a more aggressive infection (Setsu et al., 2017). 2) Challenges in antimicrobial therapy. Patients with KPLA/KP-BSI require more aggressive and broad-spectrum antibiotic therapy to treat the systemic infection. The presence of BSI may necessitate longer durations of antibiotic treatment, which can increase the risk of adverse drug reactions and the development of antibiotic resistance (**Table 4**). The selection of appropriate antibiotics can be more challenging in KPLA/KP-BSI due to the potential for multidrug-resistant strains of *Klebsiella pneumoniae* (**Table 4**). 3) Complications and secondary infections.KP-BSI can lead to complications such as metastatic infections (e.g., Pyogenic pulmonary embolism) (Chinen, 2017), which are less common in isolated KP-LA (Luo et al., 2023). Indeed, increased rate of pyogenic pulmonary embolism was observed in the group of KPLA/KP-BSI. These complications can significantly worsen the prognosis and increase the complexity of treatment. Additionally, suppurative portal vein thrombosis occurred in 5.2% of patients (higher in KPLA/KP-BSI, though not significant), which may contribute to poorer outcomes (Elkrief et al., 2024).

This study has several limitations that need to be considered. Firstly, it is a single-center retrospective study, which inherently carries the limitations of such study designs, including potential bias and confounding variables that cannot be controlled, as well as the inability to determine whether KPLA-BSI is secondary to KPLA. Secondly, the lack of bacterial virulence identification limits our understanding of the impact of specific virulence factors on the severity and outcome of KPLA and its progression to KPLA-BSI. Additionally, the small sample size of this study may restrict the generalizability of the findings to a larger population. Another limitation is the absence of prospective follow-up, preventing the assessment of KPLA’s dynamic changes and the true underlying causes of KPLA-BSI. Moreover, the prior antibiotic treatment received by some patients prior to admission may have influenced the results. Lastly, the study focused on specific risk factors while not evaluating other potential factors, such as immune status or comorbidities, which might provide a more comprehensive understanding of KPLA-BSI.

## 5 Conclusion

The development of KPLA/KP-BSI among patients with KPLA is high, at almost one third. Once KPLA is linked to sepsis, it requires vigilance for the presence of bloodstream infections; larger liver abscesses (>10 cm) are less likely to be complicated with bloodstream infections. Of note, the outcomes in patients with KPLA become worse once they are combined with KP-BSI, which needs more attention.

## Data Availability

The raw data supporting the conclusions of this article will be made available by the authors, without undue reservation.
